# Correlation analysis between auto-immunological and mutational profiles in myelodysplastic syndromes

**DOI:** 10.1007/s00011-023-01773-5

**Published:** 2023-07-28

**Authors:** Antonio Cristiano, Riccardo Belardi, Hajro Hajrullaj, Emiliano Fabiani, Giulia Falconi, Elisa Galossi, Sergio Bernardini, Maria Teresa Voso, Marzia Nuccetelli

**Affiliations:** 1 Department of Biomedicine and Prevention, University of Rome “Tor Vergata”, Via Montpellier 1, 00133 Rome, Italy; 2 Department of Experimental Medicine, University of Rome “Tor Vergata”, Rome, Italy; 3 UniCamillus-Saint Camillus International University of Health Sciences, Rome, Italy; 4 Tor Vergata University Hospital, Rome, Italy

**Keywords:** Anti-nuclear antibodies (ANA), ANA antigenic specificity, Inflammatory and autoimmune diseases, Myelodysplastic syndromes, VEXAS, Mutational profile, Auto-immunological profile

## Abstract

**Objective and design:**

Systemic-Inflammatory-Autoimmune-Diseases (SIAD) is increasingly considered in Myelodysplastic-Syndromes (MDS). In this line, we evaluated the MDS auto-immunological profile, correlating it to the mutational landscape, trying to identify a molecular-genetic trigger agent related to SIAD.

**Methods and materials:**

Eighty-one MDS were enrolled and t-NGS was performed. Anti-Nuclear-Antibodies (ANA) were tested, and ANA-antigenic-specificity was characterized by ANA-profile, ENA-screen, anti-dsDNA. Non-Hematological-Patients (NHP) and Healthy-Donors (HD) were used as controls.

**Results:**

At clinically relevant cut-off (≥ 1:160), ANA was significantly more frequent in MDS, while ANA-antigenic-specificity showed a low association rate. ANA ≥ 1:160-positive MDS showed a mutational landscape similar to ANA-negative/ANA < 1:160 MDS. No significant correlations between mutational and immunological profiles were found and *UBA1* mutations, related to VEXAS, were absent.

**Conclusions:**

Although ANA-positivity was found to be increased in MDS, the low ANA-antigenic-specificity suggests that autoantibodies didn’t recognize autoimmune-pathognomonic antigens. The lack of relationship between genetic profile and ANA-positivity, suggests that MDS genetic variants may not be the direct cause of SIAD.

**Supplementary Information:**

The online version contains supplementary material available at 10.1007/s00011-023-01773-5.

## Key message

**Table:** 

MDS patients showed an increased ANA positivity at clinically relevant cut-off.
The low ANA antigenic specificity suggests that autoantibodies didn’t recognize autoimmune-pathognomonic antigens.
Lack of correlation between mutational-landscape and ANA suggests that somatic variants aren’t involved in SIAD.

## Introduction

MyeloDysplastic Syndromes (MDS) are a heterogeneous group of myeloid neoplasms characterized by ineffective hematopoiesis and Bone Marrow (BM) dysplasia leading to peripheral blood cytopenias and increased risk of progression to Acute Myeloid Leukemia (AML) [1]. MDS are characterized by different clinical behavior based on disease specific characteristics, such as cytogenetic alterations, BM blast infiltration, recurrent somatic mutations, altered biological pathways and DNA hypermethylation. In this line, MDS diagnosis is essentially based on morphological and cytogenetic criteria, although the molecular screening through Target-Next Generation Sequencing methods (t-NGS) is indicated to better characterize the molecular profile of the disease and for a more specific prognostic classification [2]. Indeed, as recently demonstrated by the new Molecular International Prognostic Scoring System (IPSS-M), the integration of genetic profile with hematologic and cytogenetic parameters improves the risk stratification and may drive treatment choices in all MDS subgroups, also in the context of Hematopoietic Stem Cell Transplantation (HSCT) [3–5].

However, the pathophysiology of MDS onset is very complex, and based on multi-step mechanisms involving the pluripotent Hematopoietic Stem Cells (HSC), affecting their maturation and differentiation process. The involvement of the immune system and inflammation has been indicated as one of the possible driving factors contributing to MDS development and progression [6, 7]. Immune alterations have been previously associated with an increased risk of hematological disorders, but the specific role of immune dysregulation in the MDS pathogenesis and progression remains unclear [8, 9]. In particular, a genetic predisposition associated with a concomitant dysfunction of the innate immune system, autoimmune phenomena and inflammation disorders may be one of the possible causes of Systemic Inflammatory and Autoimmune Diseases (SIAD) that have been reported in 10–30% of MDS patients [10–14].

Saif et al. have divided MDS and Chronic MyeloMonocytic Leukemia (CMML) patients into 5 different groups based on SIAD manifestations: systemic vasculitis, connective tissue disorders, isolated autoimmune phenomena, immune-mediated hematologic abnormalities, and asymptomatic serologic immunologic abnormalities [15]. In this context, a French multicenter study evaluated the incidence of SIAD in a cohort of 123 MDS patients, finding vasculitis in 32% of cases, connective tissue disorders in 25%, inflammatory arthritis in 23%, neutrophilic disorders in 10% and unclassified immune disorders in 11%. The authors emphasized that the characteristics of SIAD associated with MDS would seem to differ from idiopathic autoimmune disorders and this hypothesis was also confirmed by the increased frequency of SIAD episodes that cannot be fully classified [13].

On the other hand, asymptomatic serological immunologic abnormalities, such as autoantibodies and high pro-inflammatory cytokine levels have been also reported in about 50–60% of MDS cases, confirming the presence of a possible immunologic abnormality and/or altered immune surveillance process [13, 16, 17].

The association between SIAD and MDS is increasingly considered in the overall clinical picture of this hematological patients’ group, but the prognostic impact of SIAD remains controversial. Any efforts aimed to correlate SIAD with specific MDS subentities (according to the 2016 World Health Organization, WHO and Revised International Prognostic Scoring System, IPSS-R) revealed a wide heterogeneity and a non-significant association [10, 18, 19].

VEXAS (vacuoles, E1 enzyme, X-linked, autoinflammatory, somatic) syndrome has been recently characterized as a disease with a pathogenetic mechanisms in common between MDS and SIAD. VEXAS syndrome is an autoinflammatory disease of myeloid origin resulting from somatic mutations in the *UBA1* gene [20, 21]. Most cases of MDS in association with VEXAS syndrome have a normal karyotype and low IPSS-R; furthermore, according to the 2016 WHO classification, these MDS are mainly classified as within single lineage dysplasia (MDS-MLD) and with ring sideroblast single lineage dysplasia (MDS-RS-SLD) subgroups, emphasizing the importance of SIAD screening, especially in low risk MDS patients [22, 23].

In this line, we aimed to evaluate the auto-immunological profile of MDS patients, correlating it to the mutational landscape, in order to verify whether there was a molecular genetic trigger agent related to the presence of SIAD. In particular, we assessed the frequency of ANA and their antigenic specificity, involved in connective tissue autoimmune diseases, as well as the presence of autoantibodies frequently associated with other SIAD types reported in MDS cases, such as anti-MieloPeroxidase (MPO) and anti-Proteinase-3 (PR3), involved in vasculitis, and anti-Cyclic Citrullinated Peptide (CCP3), involved in rheumatoid arthritis. Finally, we analyzed correlations between the auto-immunological and mutational profiles was performed.

## Material and methods

### Study cohorts

The study was conducted on a retrospective cohort of 81 MDS patients (pts) consecutively enrolled in the “Gruppo Romano Laziale Mielodisplasie (GROM-L)” between 2014 and 2020 and centralized by the “F. Lo-Coco” laboratory of “U.O.S.D. Diagnostica Avanzata Oncoematologica”, Policlinico Tor Vergata, Rome, Italy.

All samples were studied at the time of MDS diagnosis or before the start of any treatment. The diagnosis of myelodysplasia was established according to morphological and immunophenotypic criteria based on 2016 WHO classification, while the IPSS-R were used for prognostic stratification, grouping patients as follows: score 1 for patients with very low and low risk; score 2 for patients with intermediate risk and score 3 for patients with high and very high risk. The main clinical characteristics of MDS patients are shown in Table 1.

**Table 1:** Main clinical characteristics of the MDS patients

Patients	n = 81
Median age (range)	73 (39–93)
Sex	
Male	42 (52%)
Female	39 (48%)
WHO 2016 classification	
MDS-SLD	13 (16%)
MDS-MLD	23 (28.4%)
MDS-RS-SLD	2 (2.5%)
MDS-RS-MLD	1 (1.2%)
MDS-EB I	13 (16%)
MDS-EB II	10 (12.3%)
MDS-U	5 (6.2%)
NA	14 (17.3%)
Karyotype	
Normal	40 (49.4%)
del(5q)	2 (2.5%)
del(11q)	2 (2.5%)
del(20q)	3 (3.7%)
del(7)	1 (1.2%)
+8	2 (2.5%)
-Y	9 (11.1%)
Complex	2 (2.5%)
Other	2 (2.5%)
NA	18 (22.2%)
IPSS-R risk categories	
1—Very low and low risk	41 (50.6%)
2—Intermediate	13 (16%)
3—Highand very high	27 (33.3%)

*MDS-SLD* myelodysplastic syndrome with single lineage dysplasia, *MDS-MLD,* myelodysplastic syndrome with multilineage dysplasia, *MDS-EB* myelodysplastic syndrome with excess blasts; *RS* ring sideroblasts, *MDS-U* myelodysplastic syndrome unclassifiable, *NA* not available

Furthermore, two independent control cohorts were included in the study: a cohort of Non-Hematological Patients (NHP; n = 53) with no previous history of autoimmune disorders and particular comorbidities, such as diabetes and Monoclonal Gammopathy of Undetermined Significance (MGUS), and a cohort of Healthy Donors (HD; n = 44), whose sample was collected before vaccination against SARS-CoV-2. The main characteristics of the two control cohorts are shown in Table 2.

**Table 2:** Main clinical characteristics of the two independent control cohorts

	Non-hematological patients (NHP)	Healthy donors (HD)
Patients	n = 53	n = 44
Median age (range)	58 (18–94)	44 (22–71)
Sex		
Male	28 (53%)	19 (43%)
Female	25 (47%)	25 (57%)

For the evaluation of the auto-immunological profile, plasma samples from the three patients’ cohort were collected by centrifugation at 2500*g* for 10 min within 1 h from collection.

Bone marrow mononuclear cells (BM‐MNCs) were isolated from all MDS patients by Ficoll-Hypaque gradient centrifugation according to manufacturer's recommendations. DNA samples were extracted from BM‐MNCs collected at the time of diagnosis using the QIAamp DNA Mini Kit (Qiagen, Milan, Italy).

The study was approved by the Ethics Committee of the “University of Rome Tor Vergata” and participants gave written informed consent, according to the Declaration of Helsinki.

### Sequencing with sophia-genetics myeloid panel

Targeted-Next Generation Sequencing was performed in all MDS samples at the time of diagnosis and DNA samples were processed as previously reported [24]. The MYeloid Solution panel (MYS_1) (SOPHiA GENETICS, Saint-Sulpice, Switzerland) was used to analyze somatic mutations in 30 genes known to be frequently mutated in myeloid malignancies (10 full genes and 20 hot-spot regions). The genes list is reported in Supplementary Table 1. The resulting captured libraries were further processed on MiniSeq sequencing platform (Illumina, San Diego, California) and the generated FASTQ sequencing files were uploaded on the SOPHiA DDM platform (SOPHiA GENETICS, Saint-Sulpice, Switzerland) for detection and annotation of genomic variants (SNVs and Indels). Only mutations identified as highly or potentially pathogenic were considered for mutational analysis. The Variant Allele Frequency (VAF) cut-off for variants detection was set to 2% and the minimum coverage threshold to 1000×.

### Sanger sequencing for *UBA1* mutations

Sanger sequencing method was performed to screen mutations in the *UBA1* gene considered the pathognomonic alteration of VEXAS syndrome and not included in our t-NGS panel. Fifty ng of DNA sample were used for the first amplification reaction (Polymerase Chain Reaction, PCR) of the specific target region of the *UBA1* gene (exon 2), using primers reported in Supplementary Table 2. PCR productions were purified using GeneUP ExoSAP kit (Applied Biosystems, Waltham, USA) and 2 µl of purified products were used for subsequent Sequencing PCR following the manufacture’s protocol of BigDye^®^ terminator v3.1 cycle sequencing kit (Applied Biosystems, Waltham, USA). Sequencing reaction products were purified using the CentriSep columns (Applied Biosystems, Waltham, USA) according to the manufacturer's recommendations. Samples were diluted with 16 µl of ABI HiDi Formamide (Applied Biosystems, Waltham, USA) and resolved on ABI 3130 automated sequencer (Applied Biosystems, Waltham, USA). Sequence Scanner Software 2.0 was used to analyze the results.

### Auto-immunological profile evaluation

For the assessment of the auto-immunological profile of MDS patients, we first performed an indirect immunofluorescence assay on Human Epitelial (HEp) cells for the screening of ANA. Subsequently, ANA positive patients were characterized by three different immunoenzymatic assays to detect the antigenic specificity: ANA profile, Extractable Nuclear Antigens (ENA) screen and anti-double strand DNA (dsDNA). In addition, the possible presence of three common autoantibodies (anti-MPO, anti-PR3 and anti-CCP3) found in MDS, were evaluated by a chemiluminescence assay. The diagnostic algorithm used for immunological assessment is shown in Supplementary Fig. 1.

### Detection of anti-nuclear antibodies

The indirect immunofluorescence assay is the gold standard for the determination of antibodies directed against nuclear antigens. The “HEp-20-10 EUROPattern cell nuclei (ANA) assay” (EUROIMMUN, Lübeck, Germany) was used to screen ANA positivity in our patients’ cohort following manufacture’s protocol. In detail, after centrifugation at 2500*g* for 10 min, plasma samples were diluted 1:80 and 1:320 using a PBS-Tween solution. In case of positivity above 1:320 dilution, additional serial dilutions (up to 1:5120) were made to characterize the positivity titer. From each dilution, 30 µl of sample were deposited on the appropriate wells of kit slides and incubated for 30 min at room temperature. Slides were washed 3 times with buffer solution and 25 µl of Fluorescein-Conjugated anti-human IgG antibodies (FITC) were deposited on the wells. After 30 min, the slides were washed and read with a fluorescence microscope at 40× (EUROStar III Plus, EUROIMMUN, Lübeck, Germany). According to the guidelines, a positivity at the 1:80 dilution is considered as weak positivity (screening positivity), on the other hand, a positivity greater than or equal to 1:160 is considered as clinically relevant [25, 26].

### Identification of ANA antigenic specificity

Semiquantitative determination of the antigen specificities was performed exclusively on ANA positive plasma samples using the ANA profile, ENA screen, and anti-dsDNA immunoenzymatic assays.

The ENA screen and anti-dsDNA chemiluminescence immunoassays were performed using the QUANTA Flash ENA7^®^ (Inova Diagnostics, San Diego, USA) on the fully automated BIO-FLASH^®^ instrument (Inova Diagnostics, San Diego, USA). In details, plasma sample, magnetic particles coated with the specific antigens, and the buffer solution are combined inside a cuvette. The beads are then magnetized and washed several times until isoluminol conjugated secondary antibodies are added. The light produced from a positive reaction is measured as Relative Light Units (RLUs) by the optical system; RLUs are proportional to the amount of bound isoluminol conjugate, which is proportional to the autoantibodies concentration in the positive plasma samples. The instrument automatically develops a calibration curve that is used by the software to calculate the ng/ml values based on the RLUs values obtained for each sample.

To detect the single positivity for each autoantigen, ANA profile immunoassay was performed using the AD ANA19DBDM kit (ALPHADIA Diagnostic Products, Mont-Saint-Guibert, Belgium) on the automated “Blue Diver” instrument (Alifax, Padova, Italy). This Dot-Blot analysis consists of Strip-tests containing a positive control, a negative control, and the specific antigens spotted in triplicate on nitrocellulose membrane.

The simultaneous analysis of ANA profile, ds-DNA and ENA screen allowed us to assess the presence of different nuclear and cytoplasmic antigens commonly evaluated in autoimmune disorders, Supplementary Table 3.

### Evaluation of anti-myeloperoxidase, anti-proteinase-3 and anti-cyclic citrullinated peptide autoantibodies

Semiquantitative determination of anti-MyeloPeroxidase antibodies (anti-MPO), anti-PRoteinase-3 (anti-PR3) and anti-Cyclic Citrullinated Peptide (anti-CCP3) IgG autoantibodies were performed by QUANTA Flash^®^ assay (Inova Diagnostics, San Diego, USA), using the BIO-FLASH^®^ instrument (Inova Diagnostics, San Diego, USA). Purified MPO, PR3 and CCP3 antigens are complexed to magnetic beads. Plasma samples were diluted by the instrument and incubated at 37 °C. During incubation, the autoantibodies (anti-MPO, anti-PR3, anti-CCP3) bind the specific antigen on the magnetic beads. Following the addition of the isoluminol conjugated IgG antibody, a light reaction will be emitted, which is subsequently quantified in RLUs by the optical system of the instrument. The RLUs values are directly proportional to the autoantibodies concentration in tested plasma samples.

### Statistical analysis

A descriptive analysis of patients' biological and clinical characteristics was performed including median and range for continuous variables, and absolute and relative frequencies for categorical variables. Nonparametric tests were used to assess differences between groups (Fisher's exact test and Chi-square test, for categorical variables). For values less than 5, the Fisher's exact test and the limits for confidence intervals were used. More than two categorical groups comparison was determined by Chi-square test. All statistical comparisons were based on two-tailed tests, accepting p ≤ 0.05 as significant. All analyses were performed using GraphPad Prism (GraphPad Software, San Diego, CA).

## Results

### MDS mutational landscape

In the first step of analysis, the mutational landscape of MDS patients (n = 81) was characterized at the time of diagnosis using the MYS_1 panel by Sophia Genetics.

A total of 150 genetic variants were found (median number of variants for patient: 2, range 0–6). In particular, 15/81 pts (18.5%) showed a wild-type mutational profile, otherwise in 21/81 pts (25.9%) a single mutated gene was shown, in 23/81 pts (28.4%) two mutated genes, in 10/81 pts (12.3%) three mutated genes, in 8/81 pts (9.9%) four mutated genes, in 3/81 pts (3.7%) five mutated genes, and finally in only one case (1/81, 1.2%) six mutated genes were detected.

**Fig. 1:**
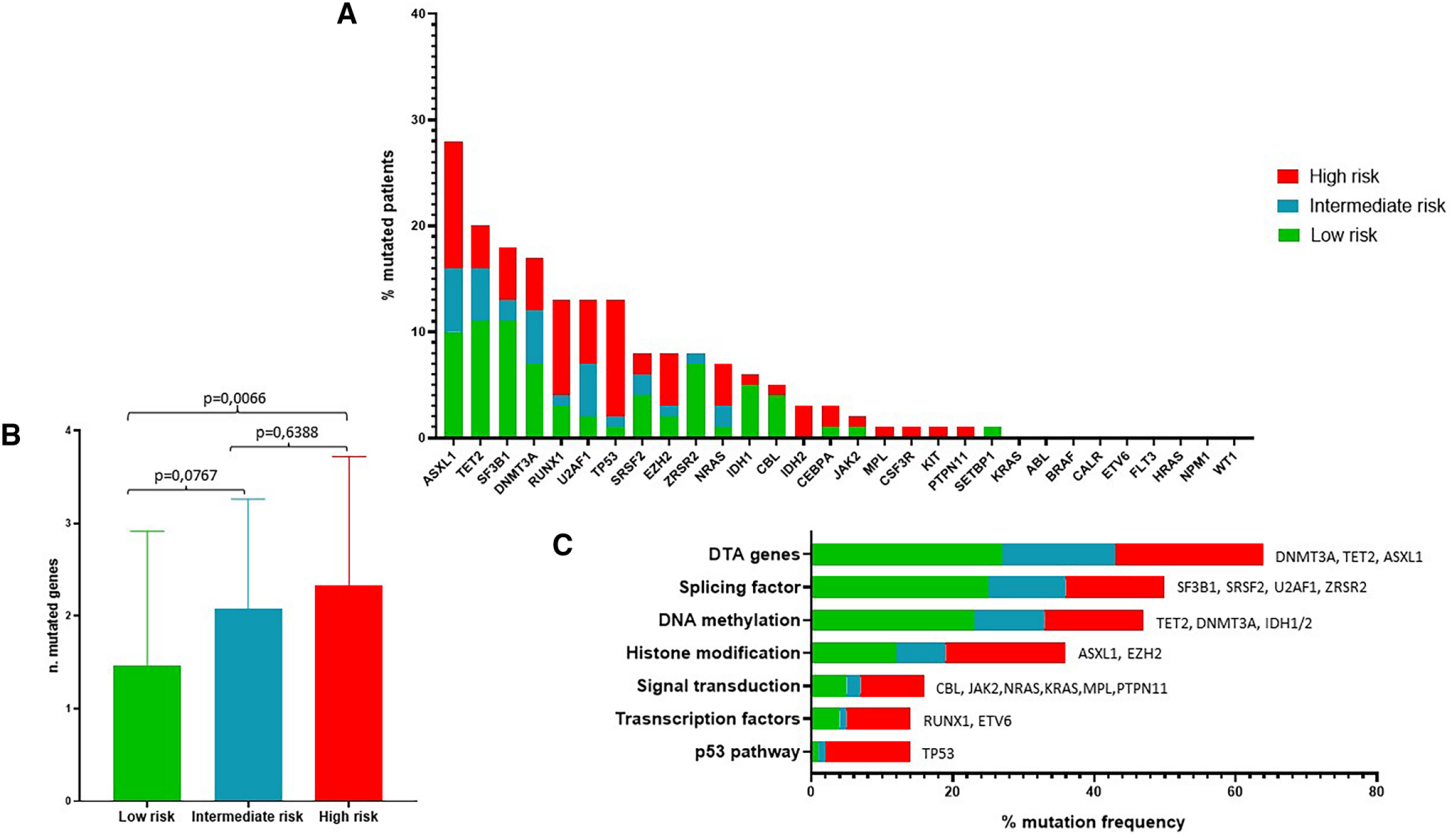
**A** Mutational landscape of MDS cohort; **B** correlation analysis between IPSS-R score category and number of mutated genes; **C** frequency of altered biological pathways in MDS cohort

The identified mutated genes were *ASXL1* (23/81 pts, 28.4%) followed by *TET2* (16/81 pts, 19.7%), *SF3B1* (15/81 pts, 18.5%), *DNMT3A* (14/81 pts, 17.3%), *U2AF1*, *RUNX1* and *TP53* (11/81 pts, 13.6%), *SRSF2*, *EZH2* and *ZRSR2* (7/81 pts, 8.6%), *NRAS* (6/81 pts, 7.4%), *IDH1* (5/81 pts, 6.2%), *CBL* (4/81 pts, 4.9%), *IDH2* and *CEBPA* (3/81 pts, 3.7%), *JAK2* (2/81 pts, 2.5%). One variant each was detected in *MPL, CSF3R*, *KIT*, *PTPN11* and *SETBP1* (1/81 pts, 1.2%), Fig. 1A. Of note, most of *TP53* mutations were found to be associated with IPSS-R high, underlying the well-established negative prognostic factor of this gene.

Results from correlation analysis between the IPSS-R prognostic score and the number of mutated genes showed a statistically significant increase in the number of mutated genes with the concomitantly increase of IPSS-R. In details, very low and low risk patients (n = 41) showed a median of one mutated gene for pts (range 0–6) vs a median of 2 mutated genes for intermediate (n = 13; range 0–4) and very high and high risk (n = 27; range 0–5) patients (p = 0.0767 and p = 0.0066, respectively), Fig. 1B.

In addition, we assessed the frequency of mutated genes according to their affiliation to a specific biological pathway and we found that the splicing factors pathway was the most commonly represented (40/81 mutated pts; 49.4%), followed by DNA methylation (38/81 pts; 46.9%), histone modification pathway (30/81 pts; 37%), signal transduction (14/81, 17.3%), transcription factors and p53 (11/81, 13.6%), Fig. 1C. To note, we also reported the frequency of DTA (*DNMT3A*, *TET2*, and *ASXL1*) genes (52/81; 64.2%), which are commonly associated to clonal hematopoiesis (CH) as possible pathogenic cause of MDS onset [27, 28], Fig. 1C.

### Anti-nuclear antibodies

ANA screening was performed by indirect immunofluorescence assay using the HEp-20–10 cells. All plasma samples were diluted and evaluate up to 1:5120 in order to more accurately establish the positivity titer. To note, positivity at 1:80 dilution is considered the screening cut-off, while positive samples ≥ 1:160 are considered clinically relevant.

**Fig. 2:**
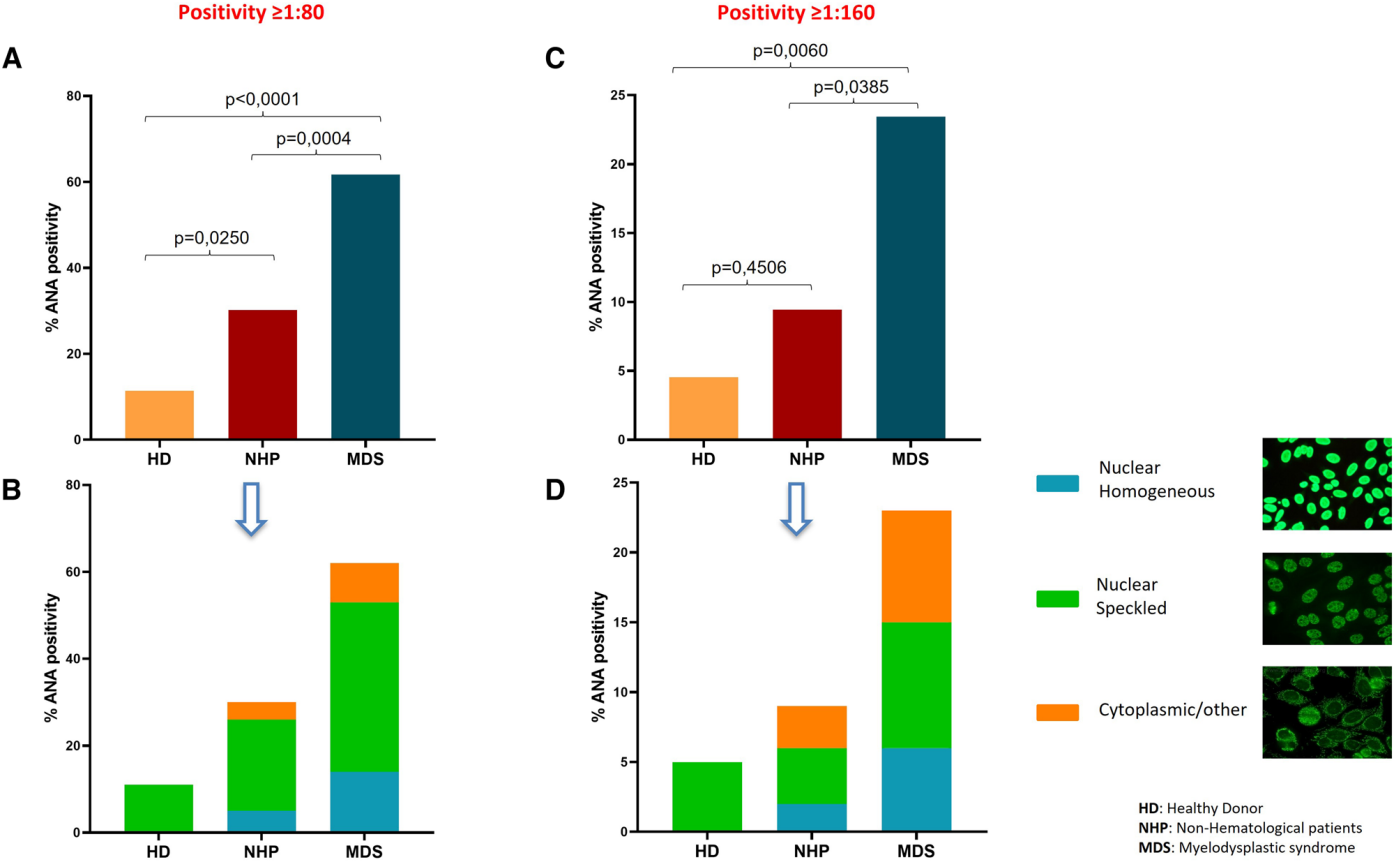
Frequency of ANA positive patients in all study cohort. **A** ANA results from 1:80 cut-off; **B** ANA results from 1:160 cut-off. **C** frequency of fluoroscopic pattern in ≥ 1:80 ANA positive patients. **D** frequency of fluoroscopic pattern in ≥ 1:160 ANA positive patients

Results showed a statistically significant difference among the 3 study cohorts, with a higher ANA positive percentage in MDS patients. In detail, based on 1:80 diluted samples, 61.7% (50/81 pts) of MDS patients presented ANA positivity, compared to 30.2% in the NHP group (16/53 pts; p = 0.0004) and 11.3% in the HD group (5/44 pts; p < 0.0001), Fig. 2A.

The same trend of data is also confirmed by adopting the clinically relevant cut-off (≥ 1:160) although with an overall lower percentage of ANA positivity. In particular, results showed 23.5% (19/81 pts) of ANA positive patients in the MDS group vs 9.4% (5/53 pts; p = 0.0385) in the NHP group and 4.5% (2/44 pts; p = 0.0060) in the HD group, Fig. 2C.

The positive samples were also evaluated by fluoroscopic pattern: at 1:80 cut-off, in MDS group 32/50 (64%) samples showed nuclear speckled patterns, 11/50 (22%) samples showed homogeneous nuclear patterns, 2/50 (4%) cytoplasmic patterns and nucleolar patterns, and 1/50 (2%) ribosomal pattern, nuclear dots pattern and nuclear membrane fluorescence pattern; in NHP patient group, 11/16 (68.7%) nuclear speckled patterns, 3/16 (18.7%) homogeneous nuclear patterns, 1/16 (6.2%) cytoplasmic pattern and 1/16 (6.2%) nucleolar pattern were recorded; in HD group 5/5 (100%) nuclear speckled patterns were observed, Fig. 2B.

Results from restrictive assessment at the clinically relevant cut-off (≥ 1:160) confirmed the prevalence of nuclear speckled patterns in the ANA positive MDS group (7/19 pts; 36.8%), followed by homogeneous nuclear pattern (5/19 pts; 26.3%), cytoplasmic pattern and nucleolar pattern (2/19 pts; 10.5%), and ribosomal pattern, nuclear dots pattern and nuclear membrane fluorescence pattern (1/19 pts; 5.3%); in NHP patients, 2 nuclear speckled patterns (2/5 pts; 40%), 1 homogeneous nuclear pattern (1/5 pts; 20%), 1 cytoplasmic pattern (1/5 pts; 20%) and 1 nucleolar pattern (1/5 pts; 20%) were observed; in the HD control group both samples were characterized by nuclear speckled patterns (2/2 pts; 100%); Fig. 2D.

### Evaluation of ANA antigenic specificity

ANA positivity is considered as a prerequisite for subsequent evaluation of the antigenic specificity by 3 different immunoenzymatic assay: dsDNA, ANA profile, and ENA screen. We evaluated all ANA positive samples in the three-study cohort.

At 1:80 cut-off, the following antigenic specificities were identified: in MDS patients (n = 50/81), ANA profile was positive in 38% of cases (19/50 pts), dsDNA and ENA screen in 8% of cases (4/50 pts), Supplementary Fig. 2; In NHP patients’ group (n = 16/53), the association rates of antigenic specificity were 18.7% (3/16 pts) for ANA profile and 6.2% (1/16 pts) for ENA screen, whilst no positivity was found with dsDNA assay, Supplementary Fig. 3; finally, in the HD group (n = 5) no antigenic positivity was found, Supplementary Fig. 4.

The antigenic specificity analysis was also performed at the clinically relevant cut-off (≥ 1:160) showing a lower antigenic correlation. In particular, we found ANA profile positivity in 9/19 pts (47.3%) while 3/19 (15.8%) MDS patients displayed ENA screen positivity and 2/19 (10.5%) dsDNA positivity, Supplementary Fig. 2. In contrast, for the NHP patients’ group only ANA profile positivity was detected in 40% (2/5 pts) of cases, Supplementary Fig. 3. No antigen specificity was found for patients belonging to the HD group (n = 2), Supplementary Fig. 4.

### Evaluation of anti-MPO, anti-PR3 and anti-CCP3 antibodies

The screening analysis for anti-MPO, anti-PR3 and anti-CCP3 autoantibodies showed positive results only in the MDS cohort; results from 1:80 diluted samples showed one anti-CCP3 positive patients (1/50 pts; 2%), and this positivity was also confirmed at ≥ 1:160 clinically relevant cut-off (1/19; 5.2%), Supplementary Fig. 5.

### Immunological profile of ANA ≥ 1:160 positive MDS patients

In the second step of our analysis, we decided to better characterize the immunological profile of ANA ≥ 1:160 positive MDS patients (19/81; 23.5%) as it is considered the clinically relevant cut-off. This patients’ group was characterized by a median age of 79 years (range 48–87 years) and the main characteristics of the study cohort are shown in Table 3.

**Table 3:** Main clinical characteristics of the ANA ≥ 1:160 positive MDS patients

Patients	n = 19
Median age (range)	79 (48–87)
Sex	
Male	8 (42.1%)
Female	11 (57.9%)
WHO 2016 classification	
MDS-SLD	3 (15.8%)
MDS-MLD	7 (36.8%)
MDS-EB I	3 (15.8%)
MDS-EB II	3 (15.8%)
NA	3 (15.8%)
Karyotype	
Normal	8 (42.1%)
del(20q)	1 (5.3%)
-Y	2 (10.5%)
Complex	2 (10.5%)
Other	2 (10.5%)
NA	4 (21.1%)
IPSS-R risk categories	
1—Very low and low risk	7 (36.8%)
2—Intermediate	6 (31.6%)
3—Highand very high	6 (31.6%)

*MDS-SLD* myelodysplastic syndrome with single lineage dysplasia, *MDS-MLD* myelodysplastic syndrome with multilineage dysplasia, *MDS-EB* myelodysplastic syndrome with excess blasts, *NA* not available

The correlation analysis between patient age and ANA positivity showed an increasing trend in ANA positivity with increasing patients’ age, Supplementary Fig. 6A: in particular, results showed 10% (2/20 pts) of ANA ≥ 1:160 positive MDS in the group aged up to 65 years compared to MDS patients older than 65 years (27.9% 17/61 pts, p = 0.1339).

In addition, dividing patients according to IPSS-R risk category, we found a statistically significant difference only comparing low risk with intermediate risk patients, Supplementary Fig. 6B; in contrast, no difference was shown when dividing patients according to WHO 2016 classification, Supplementary Fig. 6C.

Furthermore, as previously described, the evaluation of antigenic specificity showed a low association rate between ANA ≥ 1:160 positivity and antigens commonly related to autoimmune diseases, included in the tested immunoenzymatic assays: dsDNA, ENA screen and ANA profile. In particular, 10/19 (52.6%) ANA ≥ 1:160 positive patients showed no antigen specificity, Table 4.

**Table 4:** Antigenic specificity of ANA ≥ 1:160 positive MDS patients

UPN	Fluorescence pattern	ANA profile	ENA screen	dsDNA	anti-CCP3	anti-MPO	anti-PR3
5	Nucleolar 1:160	NEG	NEG	NEG	NEG	NEG	NEG
14	Homogeneous nuclear 1:160	NEG	NEG	NEG	NEG	NEG	NEG
15	Homogeneous nuclear 1:2560/ Mitotic spindle	POS (SSA/Ro 60 kD)	POS	NEG	NEG	NEG	NEG
19	Nuclear speckled 1:160	NEG	NEG	NEG	NEG	NEG	NEG
20	Homogeneous nuclear 1:160	NEG	NEG	NEG	NEG	NEG	NEG
25	Nuclear speckled 1:160	POS (SSA 52)	NEG	NEG	NEG	NEG	NEG
26	Cytoplasmic/ribosomal 1:2560	POS (SSA/Ro 52 kD/ RNA polymerase)	POS	NEG	NEG	NEG	NEG
35	Nucleolar 1:320/mitotic spindle	POS (RNA polymerase, CENP-A/B)	NEG	NEG	NEG	NEG	NEG
36	Homogeneous nuclear 1:160	POS (nucleosome)	NEG	POS	NEG	NEG	NEG
37	Nuclear speckled 1:160	POS (nucleosome, dsDNA, PCNA)	POS	POS	POS	NEG	NEG
41	Nuclear speckled 1:160	NEG	NEG	NEG	NEG	NEG	NEG
42	Cytoplasmic 1:2560	NEG	NEG	NEG	NEG	NEG	NEG
43	Cytoplasmic/cytoplasmic membrane 1:2560	NEG	NEG	NEG	NEG	NEG	NEG
48	Nuclear speckled 1:160	POS (DFS-70)	NEG	NEG	NEG	NEG	NEG
53	Homogeneous nuclear 1:160	POS (nucleosome; dsDNA)	NEG	NEG	NEG	NEG	NEG
56	Cytoplasmic 160	NEG	NEG	NEG	NEG	NEG	NEG
68	Nuclear speckled 1:160	NEG	NEG	NEG	NEG	NEG	NEG
74	Nuclear speckled 1:160	NEG	NEG	NEG	NEG	NEG	NEG
76	Nuclear dots 1:640	POS (SSA/Ro 52kD; CENP-A/B)	NEG	NEG	NEG	NEG	NEG

*POS* positive, *NEG* negative, *anti-CCP3* anti-cyclic citrullinated peptide, *anti-MPO* anti-mieloperoxidase, *anti-PR3* anti-proteinase-3 (PR3)

However, the assessment of antigen specificity by ANA profile assay had a positive result in 9/19 cases and the antigens detected were: SSA/Ro 52Kd (3/9 pts, 33.3%), Nucleosomes (3/9 pts, 33.3%), dsDNA (2/9 pts, 22.2%), CENP-A/B (2/9 pts, 22.2%), RNA polymerase (1/9 pts, 11.1%), SSA/Ro 60Kd (1/9 pts, 11.1%), PCNA (1/9 pts, 11.1%) and DFS-70 (1/9 pts, 11.1%), Table 4. The ANA profile positive patients (9/19 pts, 47.3%) showed homogeneous and nuclear speckled fluorescence patterns in 45% of cases (3/9 pts, respectively); cytoplasmic, nucleolar and nuclear dots fluorescence patterns in 11.1% of cases (1/9 pts, respectively).

In contrast, ENA screen assay results showed antigenic correlation only in 15.8% of cases (3/19 pts). For these patients, ANA fluorescence pattern analysis showed three different patterns: homogeneous, cytoplasmic and nuclear speckled. In all cases of ENA screen positivity, simultaneous positivity to ANA profile was found. Only one patient (1/3, 33.3%) was also positive to dsDNA assay. Finally, 2/19 patients (10.5%) showed positivity to dsDNA, and 1/2 pts (50%) presented a homogeneous fluorescence pattern and 1/2 pts (50%) a nuclear speckled pattern. Of note, the UPN36 patient presented simultaneous positivity to ANA and dsDNA profiling, while UPN37 patient showed positivity for all immunoenzymatic assays, Table 4.

### Correlation analysis between immunological and mutational profile of ANA ≥ 1:160 positive MDS patients

In the last part of the study, we decided to evaluate a possible correlation between mutational and immunological profiles of ANA ≥ 1:160 positive MDS patients (n = 19).

In order to rule out any possible bias, we initially performed a mutational screening analysis for the *UBA1* gene, the pathognomonic alteration recently associated with VEXAS syndrome. Since the *UBA1* gene was not included in our t-NGS panel, we designed and validated a Sanger sequencing assay. However, none of ANA ≥ 1:160 positive MDS patients showed mutations in the *UBA1* gene, confirming that no VEXAS patients were included in the study cohort.

**Fig. 3:**
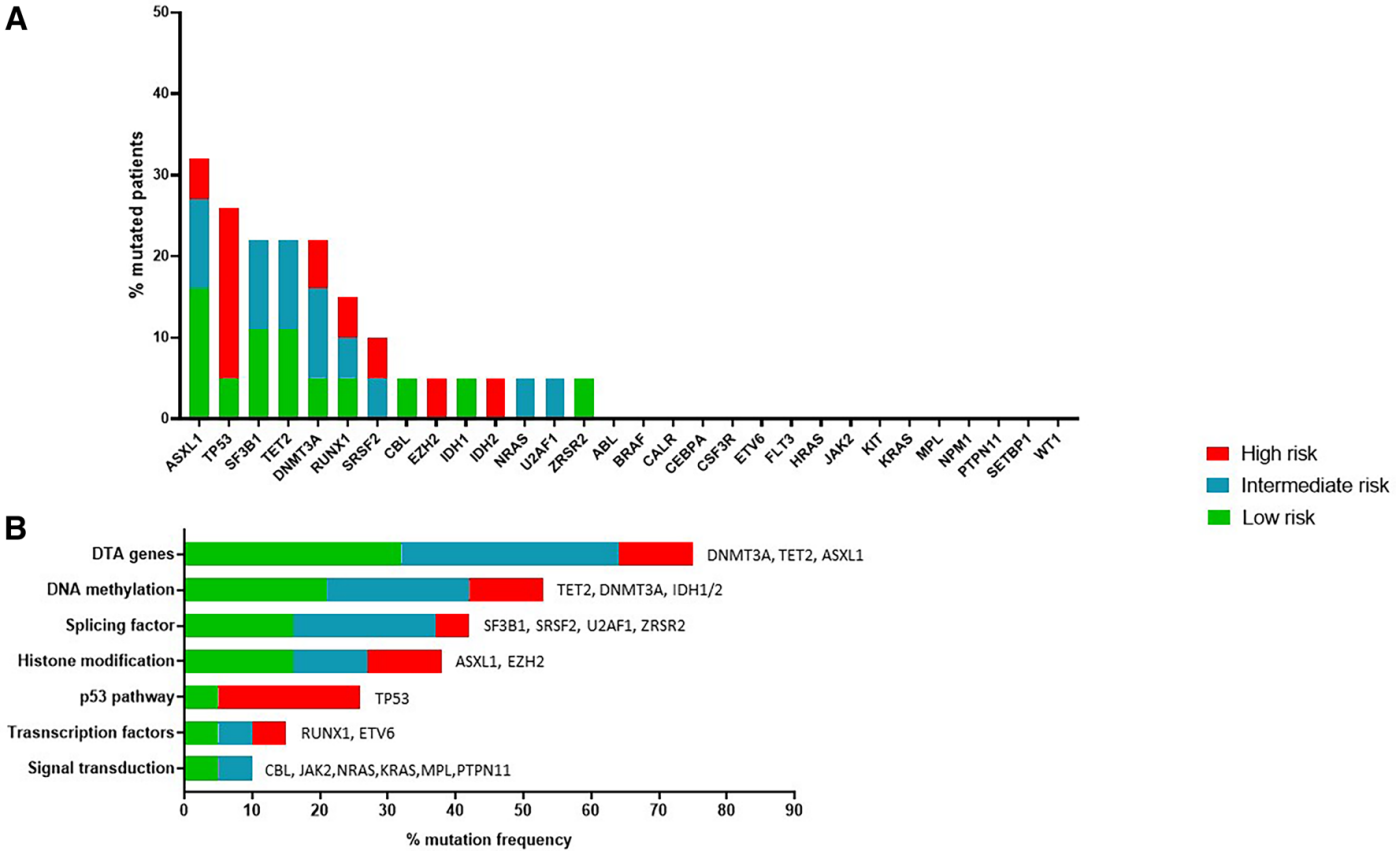
**A** Mutational landscape of ANA ≥ 1:160 positive MDS; **B** frequency of altered biological pathway in ANA ≥ 1:160 positive MDS

After this control analysis, we evaluated the mutational landscape of ANA ≥ 1:160 positive MDS patients. The obtained results were similar to the entire MDS cohort, except for an increased number of mutations in *TP53* gene (26.3%, 5/19 pts vs 13.6% 11/81 pts, p = 0.1179), Fig. 3A.

*ASXL1* was confirmed as the most frequently mutated gene (6/19 pts, 31.6%), followed by *TP53* (5/19 pts, 26.3%), *TET2*, *DNMT3A* and *SF3B1* (4/19 pts, 21.1%), *RUNX1* (3/19 pts, 15.8%), *SRSF2* (2/19 pts, 10.5%) and finally *CBL, EZH2*, *IDH1*, *IDH2*, *NRAS*, *U2AF1*, *ZRSR2* (1/19 pts, 5.3%); Fig. 3A.

**Fig. 4:**
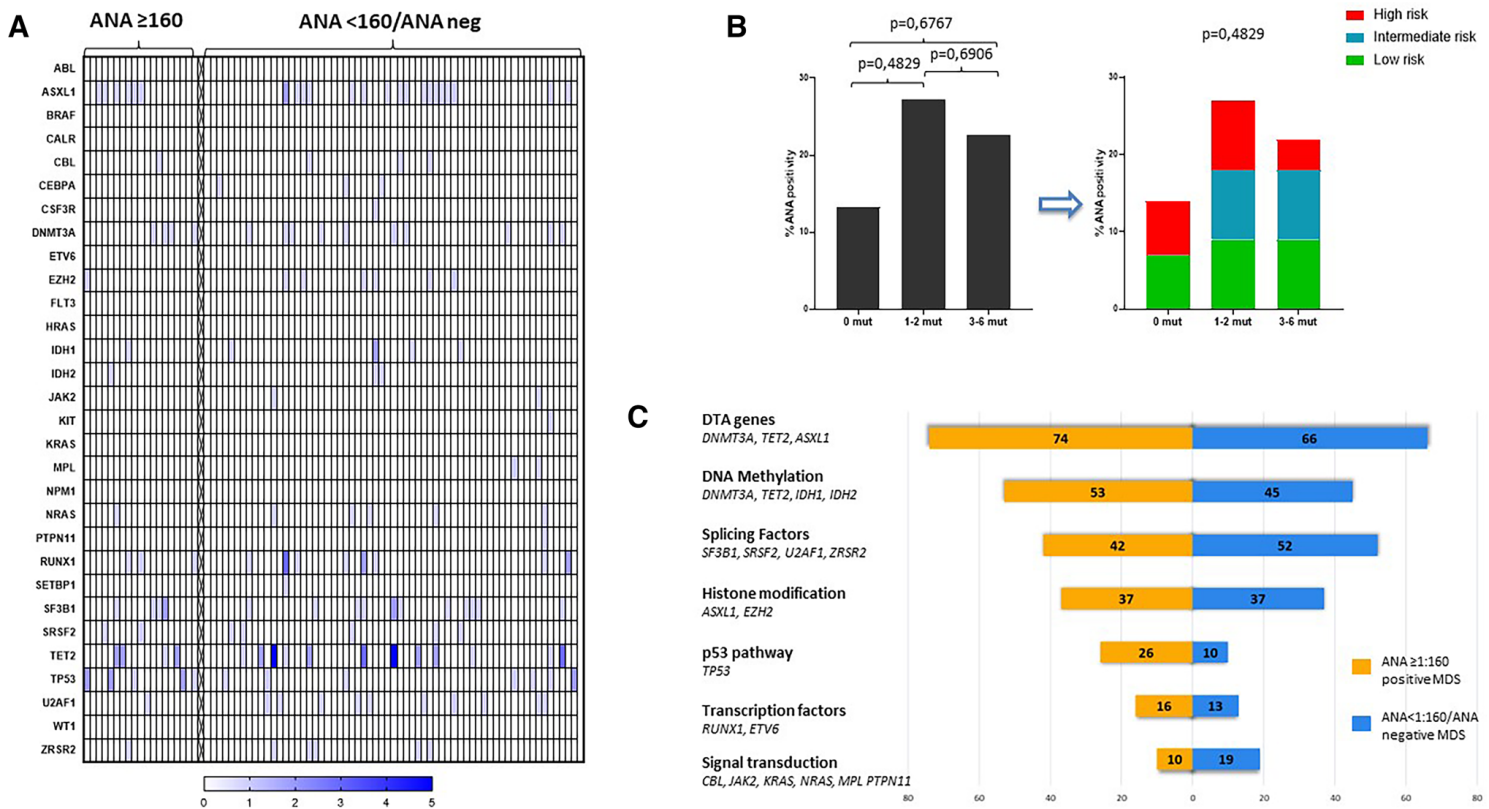
**A** Heatmap analysis comparing ANA ≥ 1:160 positive MDS and MDS group negative for ANA or with ANA < 1:160; **B** Correlation analysis between ANA positivity ≥ 1:160 and number of genetic variants identified in the MDS cohort; **C** frequency of altered biological pathway comparing ANA ≥ 1:160 positive MDS and MDS negative for ANA or with ANA < 1:160

The identified gene variants were grouped according to their biological pattern. Results showed that the most representative pathway was the DNA methylation pathway (52.6%; 10/19 pts), followed by the splicing machinery pathway (42.1%, 8/19 pts), the pathway involved in histone modifications (36.9%, 7/19 pts), the p53 pathway (26.3%, 5/19 pts), the signal transduction pathway (15.8%, 3/19 pts) and finally the pathway including transcription factors (10.5%, 2/19 pts), Fig. 3B. To note, we also evaluated the involvement of DTA genes and results showed that 14/19 (73.7%) patients had at least one variant in the three genes, Fig. 3B.t-NGS results showed a total of 35 variants identified in the MDS group with ANA ≥ 1:160 (n = 19), with a median of 2 mutated genes per patient (range 0–4), compared with 115 variants identified in the MDS group with ANA negative or with ANA < 1:160 (n = 62; median of 2 mutations per pts; range 0–6). Graphical analysis by heatmap method showed no significant differences between the two groups analyzed, as confirmed by the Chi-square test, which returned a p-value that was not statistically significant, Fig. 4A.

In the second stage of mutational analysis, we performed a correlation analysis between ANA positivity ≥ 1:160 and the number of genetic variants identified in the MDS cohort, also dividing MDS patients into IPSS-R categories. In particular, results showed that 2/15 pts (13.3%) with wild-type mutational profile had ANA positivity ≥ 1:160; in the group of patients with 1–2 mutated genes, the percentage of ANA ≥ 1:160 positivity increased to 27.3% (12/44 pts), and finally, we found 5/22 pts (22.7%) positive to ANA ≥ 1:160 in the group of patients with 3–6 mutated genes, Fig. 4B.

In addition, we tried to compare the frequency of the most mutated genes between the two groups of ANA ≥ 1:160 MDS patients and MDS patients with ANA negativity or ANA < 1:160 but no statistically significant results were found, Supplementary Fig. 7.

Finally, we evaluated the possible involvement of specific biological pathways in the ANA ≥ 1:160 positive MDS patients (n = 19) comparing the obtained results with MDS patients with ANA negativity or ANA < 1:160 (n = 62). For this analysis, we also considered the possible involvement of DTA genes, Fig. 4C and Supplementary Table 4.

## Discussion

Systemic inflammatory disease and autoimmunity represent a dysregulation of immune system and immune tolerance mechanism. To date, there is a growing interest on the role of immune system as a trigger factor in the context of neoplasm onset, promoting the development of a permissive microenvironment for tumor cells proliferation [29, 30].

In this context, SIAD have been associated with an increased risk of developing hematological disorders in both lymphoid and myeloid neoplasm [8]. The dysregulation of immunological environment and associated inflammatory conditions could play an important role in the pathogenesis and progression of hematological disease, although the specific relationship between the two pathological phenomena is not yet understood.

The contextual presence of immune alterations and hematological disorders remains a chicken-or-egg causality dilemma. In several different cases, the immunologic disorders have been described before the hematological disorders diagnosis, whilst in other cases the immune alterations have been reported after hematological neoplasm onset or during treatment. In this line, immunosuppressive therapies such as immune checkpoint inhibitors, INF-alpha, corticosteroids, monoclonal antibodies and cytotoxic drugs, may induce immunological perturbations and/or rheumatic manifestations during the management of hematological patients [31–33].

Despite the heterogeneity of reported data, the presence of systemic inflammatory and autoimmune disorders have been reported in about 10–30% of MDS cases, with an increased frequency in elderly patients [34–36].

SIAD in MDS patients include several different manifestations: systemic vasculitis, connective tissue disorders, immuohematological disorders and serological abnormalities [13, 37]. It should be emphasized that the divergences of the reported cases may be due to different clinical and diagnostic classification of inflammatory and autoimmune phenomena, as well as the different evaluation of laboratory data. Moreover, inflammatory and autoimmune disorders represent a large and heterogeneous group of diseases, which can manifest clinically in many different ways, in some cases even phenotypically silent, with laboratory abnormalities detectable only on analysis of serum or plasma samples.

In this grey zone, the aim of our study was to evaluate the correlations between the immunological and mutational profiles in MDS patients.

Thought t-NGS analysis, our MDS cohort showed a mutational profile in line with previously reported data, with DTA genes as the most frequently mutated genes [38, 39]. A direct correlation between the number of somatic mutations and the IPSS-R risk category was found, underscoring how patients with higher IPSS-R could be characterized by a greater genomic instability.

On the other hand, the characterization of immunological profile performed on both cut-offs (1:80 and ≥ 1:160) showed a statistically increase of ANA positivity in MDS patients compared to the NHP and HD control groups. It should be noted that a significant difference was found between the HD and NHP groups considering the 1:80 cut-off; although the NHP group was represented by non-hematologic patients with no clinical history of autoimmune disease, they are still "pathological" subjects, enrolled in our center for other different clinical conditions and may be characterized by inflammatory profile that could be responsible for a non-specific ANA positivity. This hypothesis is partly confirmed by the absence of statistically significant differences between the two control groups at the ≥ 1:160 cut-off.

The reduction of ANA positive MDS patients at the clinically relevant cut-off of ≥ 1:160 suggest that the presence of anti-nuclear antibodies may be due not to a distinct autoimmune condition but rather to a general state of inflammation and immunological disorders, which could induce the production of different types of autoantibodies unable to recognize self-antigens. This hypothesis is partially confirmed by the low antigenic specificity; indeed, only 9/19 ANA ≥ 1:160 positive MDS patients had a positive result in the immunoenzymatic assays commonly used for the evaluation of the main ANA targeted antigens.

To note, it should be pointed out that in the tested immunoenzymatic assays there are self-antigens that are normally evaluated in the context of autoimmune diseases, therefore, this does not exclude the possibility that ANA in MDS patients may have a different antigenic target not included in the performed tests and which could be part of the pool of Tumor Associated Antigens (TAAs).

In the ANA ≥ 1:160 positive MDS group, a trend of increase ANA positivity with increasing patient’s age was observed, suggesting a possible greater involvement of immune system dysfunction in older patients. This data is also underscored by an increase in the median age of ANA ≥ 1:160 positive MDS patients compared to the entire MDS cohort (79 vs 73 years, respectively, p = 0.1339).

The stratification of ANA ≥ 1:160 positive MDS according to the 2016 WHO classification and IPSS-R score did not show a statistically significant association between ANA positivity and a specific MDS subgroup, although patients with intermediate risk showed the higher frequency of ANA positivity. The lack of patient stratification may again confirm the strong heterogeneity that characterizes both MDS and SIAD, complicating the diagnostic and prognostic classification of patients characterized by both hematological and immunological disorders. In this line, it is also difficult to establish a specific treatment strategy which may include both hematological and inflammatory/autoimmune treatments.

Finally, in the last part of our study, the possibility that somatic mutations could also affect the immunological profile of ANA ≥ 1:160 positive MDS was evaluated. The recent introduction of patients with VEXAS syndrome as a unique and distinct entity of patients with hematologic disorders in association with inflammatory/autoimmune phenomena, prompted us to perform a Sanger sequencing analysis to be able to exclude mutations in the *UBA1* gene, which are considered as pathognomonic alterations of VEXAS syndrome. The entire cohort of ANA ≥ 1:160 positive MDS, did not show *UBA1* mutations.

Results from t-NGS performed in ANA ≥ 1:160 positive MDS group showed a mutational landscape similar to all MDS cohort, except for an increased frequency of mutations in the *TP53* gene. These data are also confirmed by the comparative analysis of the main involved biological pathways, that showed few differences between the entire MDS cohort and the ANA ≥ 1:160 positive MDS group, summarized with the inversion between the DNA methylation pathway, which was found to be more frequently mutated in the ANA ≥ 1:160 positive MDS, and the splicing factors pathway, in addition with the increased frequency of p53 pathway.

After the characterization of the mutational profile by t-NGS, we tried to test whether there was a correlation between the mutational profile and the frequency of ANA ≥ 1:160 positivity. Results did not show a statistically significant increase of ANA positivity in relation to a specific genetic variant and/or biological pathway. These data could confirm that the genetic variants responsible for the hematologic disorder may not also be the direct cause of the inflammatory/autoimmune disorders, suggesting other possible trigger factors for the immunologic disfunction.

In conclusion, although a statistically increased presence of anti-nuclear antibodies was found in our MDS cohort, the low antigenic specificity may suggest how these autoantibodies are not directed against pathognomonic antigens of autoimmune disorders, but rather against different proteins that might be associated to inflammatory cross-reactions and systemic inflammation phenomena, typically found in patients with hematologic disorders, or toward tumor-associated antigens not yet identified in the context of MDS. The concomitant study of autoimmunity, deep clinical phenotype and MDS specific features will shed light on the role of autoantigens in MDS pathobiology.

## Supplementary Information

Below is the link to the electronic supplementary material.

Supplementary file1 (DOCX 311 KB)

## Data Availability

Sequencing data will be shared by the corresponding author upon request.

## References

[1] Arber DA, Orazi A, Hasserjian R (2016). The 2016 revision to the World Health Organization classification of myeloid neoplasms and acute leukemia. Blood. 2016;127(20):2391-2405. Blood.

[2] Malcovati L, Hellström-Lindberg E, Bowen D, Adès L, Cermak J, Del Cañizo C, Della Porta MG, Fenaux P, Gattermann N, Germing U (2013). Diagnosis and treatment of primary myelodysplastic syndromes in adults: recommendations from the European LeukemiaNet. Blood.

[3] Bernard E, Tuechler H, Greenberg PL, Hasserjian RP, Ossa JEA, Nannya Y, Devlin SM, Creignou M, Pinel P, Monnier L (2022). Molecular international prognostic scoring system for myelodysplastic syndromes. NEJM Evid.

[4] Bersanelli M, Travaglino E, Meggendorfer M, Matteuzzi T, Sala C, Mosca E, Chiereghin C, Di Nanni N, Gnocchi M, Zampini M (2021). Classification and personalized prognostic assessment on the basis of clinical and genomic features in myelodysplastic syndromes. J Clin Oncol Off J Am Soc Clin Oncol.

[5] Lindsley RC, Saber W, Mar BG, Redd R, Wang T, Haagenson MD, Grauman PV, Hu Z-H, Spellman SR, Lee SJ (2017). Prognostic mutations in myelodysplastic syndrome after stem-cell transplantation. N Engl J Med.

[6] Kristinsson SY, Björkholm M, Hultcrantz M, Derolf ÅR, Landgren O, Goldin LR (2011). Chronic immune stimulation might act as a trigger for the development of acute myeloid leukemia or myelodysplastic syndromes. J Clin Oncol Off J Am Soc Clin Oncol.

[7] Gañán-Gómez I, Wei Y, Starczynowski DT, Colla S, Yang H, Cabrero-Calvo M, Bohannan ZS, Verma A, Steidl U, Garcia-Manero G (2015). Deregulation of innate immune and inflammatory signaling in myelodysplastic syndromes. Leukemia.

[8] Barcellini W, Giannotta JA, Fattizzo B (2021). Autoimmune complications in hematologic neoplasms. Cancers (Basel)..

[9] Anderson LA, Pfeiffer RM, Landgren O, Gadalla S, Berndt SI, Engels EA (2009). Risks of myeloid malignancies in patients with autoimmune conditions. Br J Cancer.

[10] De Hollanda A, Beucher A, Henrion D, Ghali A, Lavigne C, Lévesque H, Hamidou M, Subra JF, Ifrah N, Belizna C (2011). Systemic and immune manifestations in myelodysplasia: a multicenter retrospective study. Arthritis Care Res. (Hoboken).

[11] Wolach O, Stone R (2016). Autoimmunity and inflammation in myelodysplastic syndromes. Acta Haematol.

[12] Glenthøj A, Ørskov AD, Hansen JW, Hadrup SR, O’Connell C, Grønbæk K (2016). Immune mechanisms in myelodysplastic syndrome. Int J Mol Sci.

[13] Mekinian A, Grignano E, Braun T, Decaux O, Liozon E, Costedoat-Chalumeau N, Kahn J-E, Hamidou M, Park S, Puéchal X (2016). Systemic inflammatory and autoimmune manifestations associated with myelodysplastic syndromes and chronic myelomonocytic leukaemia: a French multicentre retrospective study. Rheumatology (Oxford).

[14] Enright H, Miller W (1997). Autoimmune phenomena in patients with myelodysplastic syndromes. Leuk Lymphoma.

[15] Saif MW, Hopkins JL, Gore SD (2002). Autoimmune phenomena in patients with myelodysplastic syndromes and chronic myelomonocytic leukemia. Leuk Lymphoma.

[16] Giannouli S, Voulgarelis M (2014). A comprehensive review of myelodysplastic syndrome patients with autoimmune diseases. Expert Rev Clin Immunol.

[17] Wang C, Yang Y, Gao S, Chen J, Yu J, Zhang H, Li M, Zhan X, Li W (2018). Immune dysregulation in myelodysplastic syndrome: clinical features, pathogenesis and therapeutic strategies. Crit Rev Oncol Hematol.

[18] Giannouli S, Voulgarelis M, Zintzaras E, Tzioufas AG, Moutsopoulos HM (2004). Autoimmune phenomena in myelodysplastic syndromes: a 4-yr prospective study. Rheumatology (Oxford).

[19] Komrokji RS, Kulasekararaj A, Al Ali NH, Kordasti S, Bart-Smith E, Craig BM, Padron E, Zhang L, Lancet JE, Pinilla-Ibarz J (2016). Autoimmune diseases and myelodysplastic syndromes. Am J Hematol.

[20] Grayson PC, Patel BA, Young NS (2021). VEXAS syndrome. Blood.

[21] Beck DB, Ferrada MA, Sikora KA, Ombrello AK, Collins JC, Pei W, Balanda N, Ross DL, Ospina Cardona D, Wu Z (2020). Somatic mutations in UBA1 and severe adult-onset autoinflammatory disease. N Engl J Med.

[22] Georgin-Lavialle S, Terrier B, Guedon AF, Heiblig M, Comont T, Lazaro E, Lacombe V, Terriou L, Ardois S, Bouaziz J-D (2022). Further characterization of clinical and laboratory features in VEXAS syndrome: large-scale analysis of a multicentre case series of 116 French patients. Br J Dermatol.

[23] Comont T, Heiblig M, Rivière E, Terriou L, Rossignol J, Bouscary D, Rieu V, Le Guenno G, Mathian A, Aouba A (2022). Azacitidine for patients with vacuoles, E1 enzyme, X-linked, autoinflammatory, somatic syndrome (VEXAS) and myelodysplastic syndrome: data from the French VEXAS registry. Br J Haematol.

[24] Fabiani E, Cicconi L, Nardozza AM, Cristiano A, Rossi M, Ottone T, Falconi G, Divona M, Testi AM, Annibali O (2021). Mutational profile of ZBTB16-RARA-positive acute myeloid leukemia. Cancer Med.

[25] Tan EM, Feltkamp TE, Smolen JS, Butcher B, Dawkins R, Fritzler MJ, Gordon T, Hardin JA, Kalden JR, Lahita RG (1997). Range of antinuclear antibodies in “healthy” individuals. Arthritis Rheum.

[26] Satoh M, Vázquez-Del Mercado M, Chan EKL (2009). Clinical interpretation of antinuclear antibody tests in systemic rheumatic diseases. Mod Rheumatol.

[27] Jaiswal S, Fontanillas P, Flannick J, Manning A, Grauman PV, Mar BG, Lindsley RC, Mermel CH, Burtt N, Chavez A (2014). Age-related clonal hematopoiesis associated with adverse outcomes. N Engl J Med.

[28] Genovese G, Kähler AK, Handsaker RE, Lindberg J, Rose SA, Bakhoum SF, Chambert K, Mick E, Neale BM, Fromer M (2014). Clonal hematopoiesis and blood-cancer risk inferred from blood DNA sequence. N Engl J Med.

[29] Barcellini W, Fattizzo B (2021). Immune phenomena in myeloid neoplasms: an “egg or chicken” question. Front Immunol.

[30] Turesson C, Matteson EL (2013). Malignancy as a comorbidity in rheumatic diseases. Rheumatology (Oxford).

[31] Raanani P, Ben-Bassat I (2002). Immune-mediated complications during interferon therapy in hematological patients. Acta Haematol.

[32] Kichloo A, Albosta M, Dahiya D, Guidi JC, Aljadah M, Singh J, Shaka H, Wani F, Kumar A, Lekkala M (2021). Systemic adverse effects and toxicities associated with immunotherapy: a review. World J Clin Oncol.

[33] Alizadeh M, Safarzadeh A, Hoseini SA, Piryaei R, Mansoori B, Hajiasgharzadeh K, Baghbanzadeh A, Baradaran B (2020). The potentials of immune checkpoints for the treatment of blood malignancies. Crit Rev Oncol Hematol.

[34] Fraison JB, Mekinian A, Braun T, Grignano E, Adès L, Brechignac S, Gardin C, Bourgarit-Durand A, Chollet-Martin S, Nicaise-Roland P (2015). 279 Frequency of autoantibodies (AAB) in MDS with and without clinical autoimmune disorders (AID). Leuk Res.

[35] Mekinian A, Braun T, Decaux O, Falgarone G, Toussirot E, Raffray L, Omouri M, Gombert B, De Wazieres B, Buchdaul A-L (2014). Inflammatory arthritis in patients with myelodysplastic syndromes: a multicenter retrospective study and literature review of 68 cases. Medicine (Baltimore).

[36] Castro M, Conn DL, Su WP, Garton JP (1991). Rheumatic manifestations in myelodysplastic syndromes. J Rheumatol.

[37] Grignano E, Jachiet V, Fenaux P, Ades L, Fain O, Mekinian A (2018). Autoimmune manifestations associated with myelodysplastic syndromes. Ann Hematol.

[38] Papaemmanuil E, Gerstung M, Malcovati L, Tauro S, Gundem G, Van Loo P, Yoon CJ, Ellis P, Wedge DC, Pellagatti A (2013). Clinical and biological implications of driver mutations in myelodysplastic syndromes. Blood.

[39] Haferlach T, Nagata Y, Grossmann V, Okuno Y, Bacher U, Nagae G, Schnittger S, Sanada M, Kon A, Alpermann T (2014). Landscape of genetic lesions in 944 patients with myelodysplastic syndromes. Leukemia.

