# Supplementary material for: Correlation analysis between auto-immunological and mutational profiles in myelodysplastic syndromes
Source: Inflamm Res. 2023 Jul 28;72(8):1695–707. doi: 10.1007/s00011-023-01773-5 (PMC10499973; doi:10.1007/s00011-023-01773-5)

**Supplementary Tables**

**Supplementary Table 1.** Genes list of MYeloid Solution (MYS_1) panel by Sophia Genetics

| **Gene** | **Target region**  **(exon)** | **Gene** | **Target region**  **(exon)** | **Gene** | **Target region**  **(exon)** |
| --- | --- | --- | --- | --- | --- |
| *ABL* | 4-9 | *FLT3* | 13-15 and 20 | *PTPN11* | 3,7-13 |
| *ASXL1* | 9,11,12 | *HRAS* | 2,3 | *RUNX1* | all |
| *BRAF* | 15 | *IDH1* | 4 | *SETBP1* | 4 |
| *CALR* | 9 | *IDH2* | 4 | *SF3B1* | 10-16 |
| *CBL* | 8,9 | *JAK2* | all | *SRSF2* | 1 |
| *CEBPA* | all | *KIT* | 2,8-11,13,17,18 | *TET2* | all |
| *CSF3R* | all | *KRAS* | 2,3 | *TP53* | all |
| *DNMT3A* | all | *MPL* | 10 | *U2AF1* | 2,6 |
| *ETV6* | all | *NPM1* | 10,11 | *WT1* | 6-10 |
| *EZH2* | all | *NRAS* | 2,3 | *ZRSR2* | all |

**Supplementary Table 2.** Sequences of Forward and Reverse primers for *UBA1* gene amplification

| **Primers** | **Sequence (5’🡪3’)** |
| --- | --- |
| UBA1_Fw (P831) | CCCCTCTTTGCTGTAAAATG |
| UBA1_Rv (P832) | CTCATGGCCCAACACATACC |

**Supplementary Table 3.** List of specific antigens including in ENA screen, ANA profile and dsDNA

| **Immunoenzymatic assay** | **Nuclear and cytoplasmic antigens** |
| --- | --- |
| **ENA screen** (QUANTA Flash ENA7) | Sm, RNP, Ro60 (SS-A), Ro52/TRIM21, SS-B (La), Scl-70, Jo-1 |
| **ANA profile** (AD ANA19DBDM) | Nucleosoma, dsDNA, istoni, Sm, U1-RNP, Sm/RNP, SSA/Ro60Kd, SSA/Ro52Kd, SSB, Scl-70, RNA polimerasi III, Ku, PM-Scl100, MI-2, Jo-1, CENP-A/B, PCNA, Ribosoma P0, DFS-70 |
| **dsDNA** | Double strand-DNA |

**Supplementary Table 4.** Analysis of altered biological pathway in ANA≥160 positive MDS vs ANA neg/ANA <1:160 MDS patients

| **Pathways** | **ANA pos ≥1:160** | | **ANA neg/ANA <1:160** | | **p-value** |
| --- | --- | --- | --- | --- | --- |
|  |  |  |  |  |  |
|  | **Mutated patients** | **Wild-type patients** | **Mutated patients** | **Wild-type patients** |  |
| **DTA genes** | 14 | 5 | 41 | 21 | 0,5888 |
| **DNA methylation** | 10 | 9 | 28 | 34 | 0,5681 |
| **Splicing factor** | 8 | 11 | 32 | 30 | 0,4683 |
| **Histone modification** | 7 | 12 | 23 | 39 | 0,984 |
| **p53 pathway** | 5 | 14 | 6 | 56 | 0,1179 |
| **Trasnscription factors** | 3 | 16 | 8 | 54 | 0,7139 |
| **Signal transduction** | 2 | 17 | 12 | 50 | 0,5013 |

**Supplementary Figures**

**Supplementary Figure 1.** Diagnostic algorithm used for auto-immunological profile evaluation


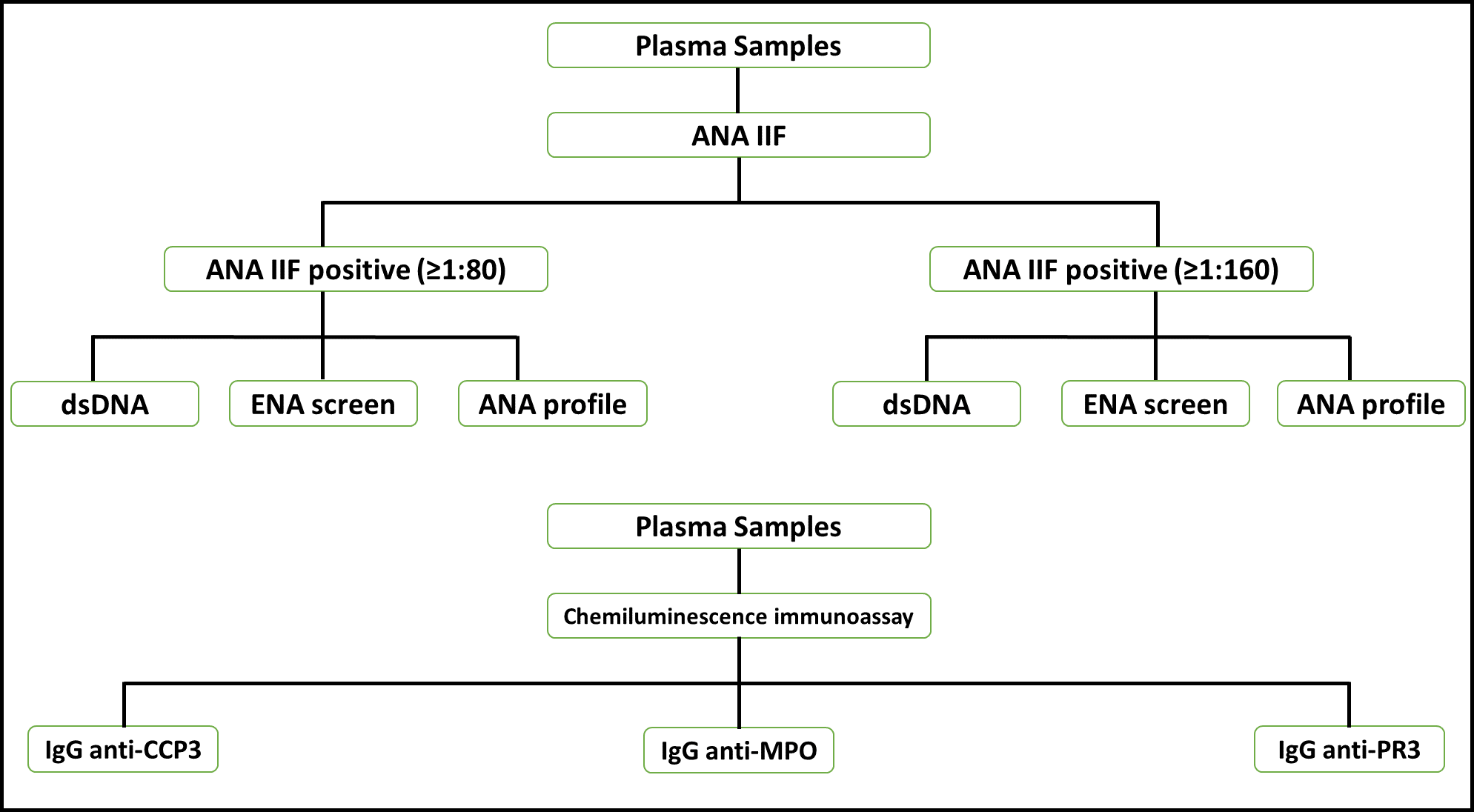


**Supplementary Figure 2.** ANA antigenic specificity in Myelodysplastic syndrome patients’ cohort


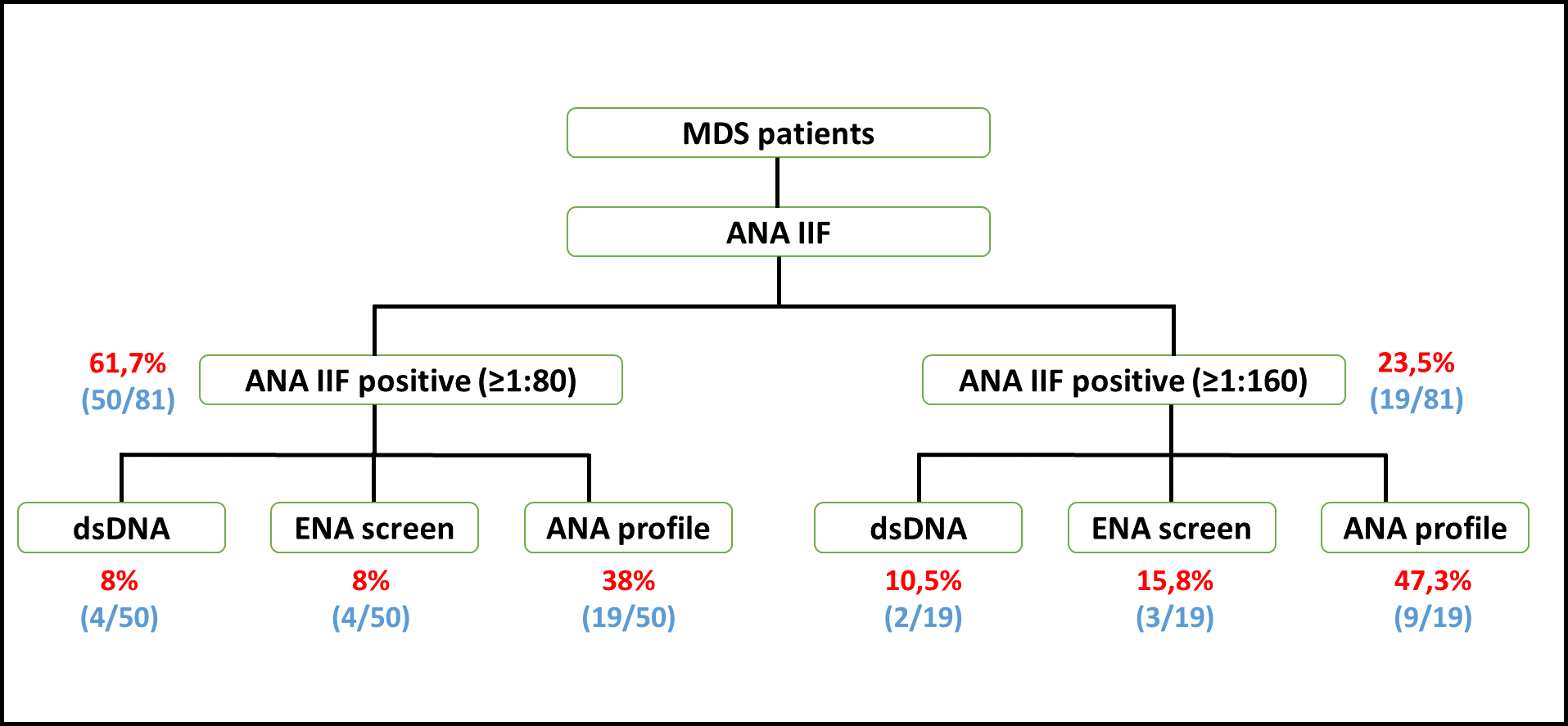


**Supplementary Figure 3.** ANA antigenic specificity in Non-Hematological patients’ cohort


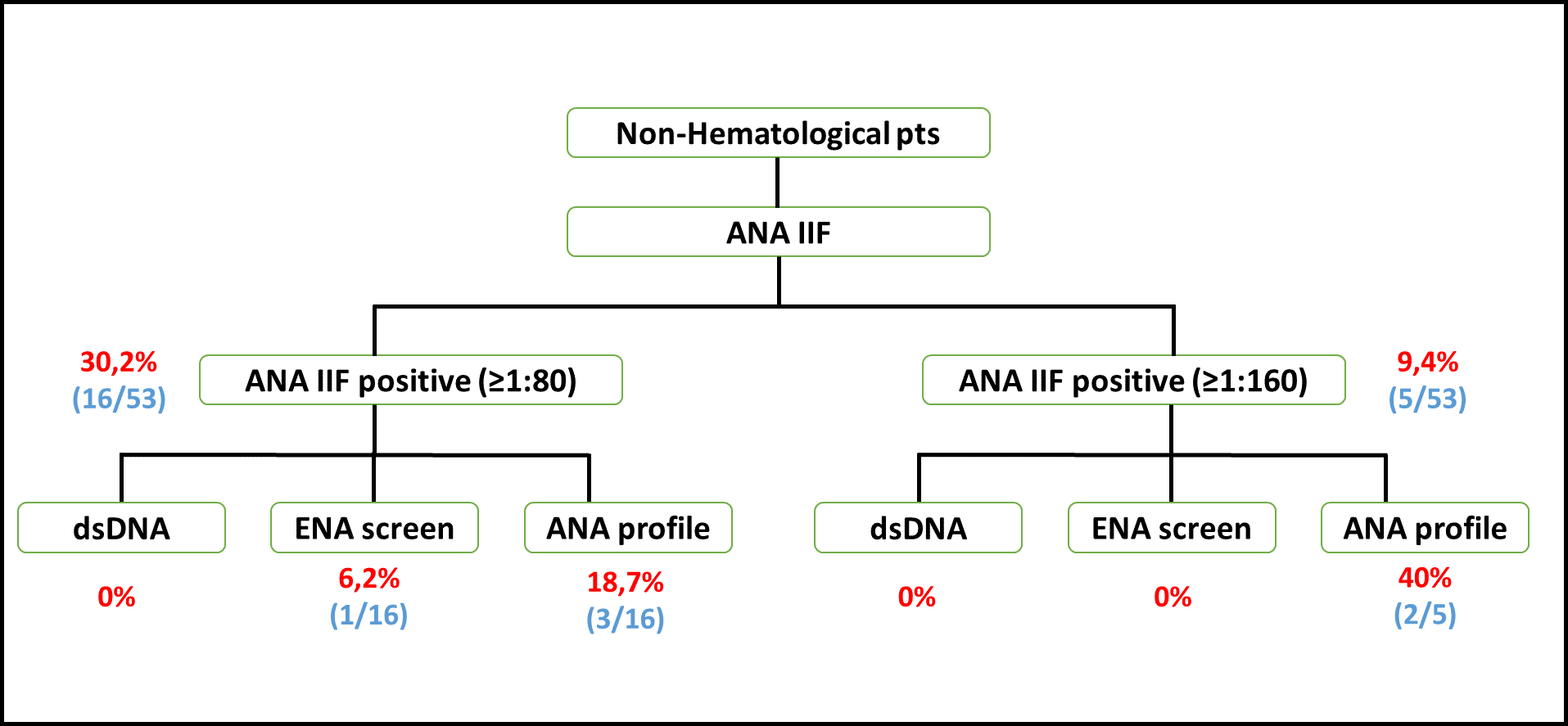


**Supplementary Figure 4.** ANA antigenic specificity in Healthy Donor cohort


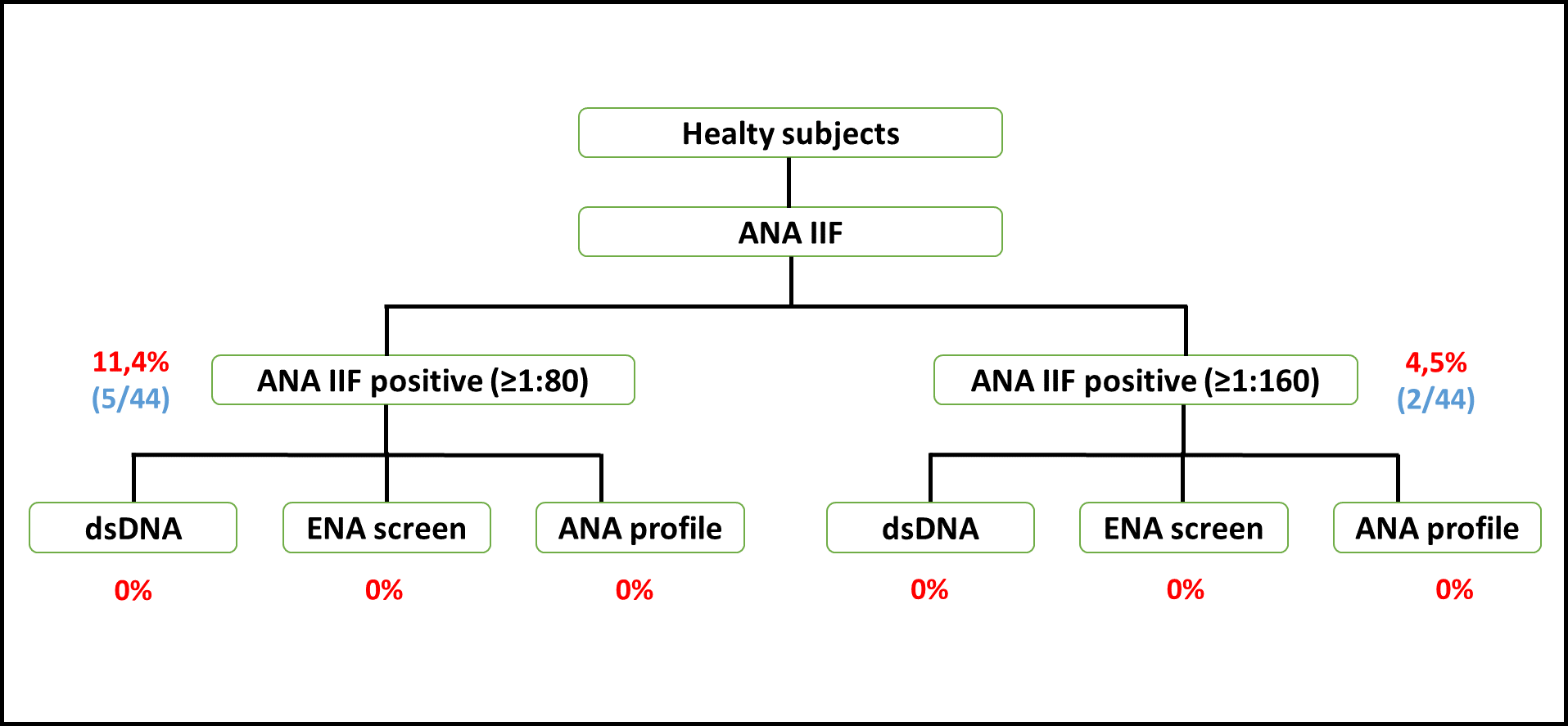


**Supplementary Figure 5.** Results of anti-MyeloPeroxidase, anti-PRoteinase-3 and anti-Cyclic Citrullinated Peptide autoantibodies in MDS cohort


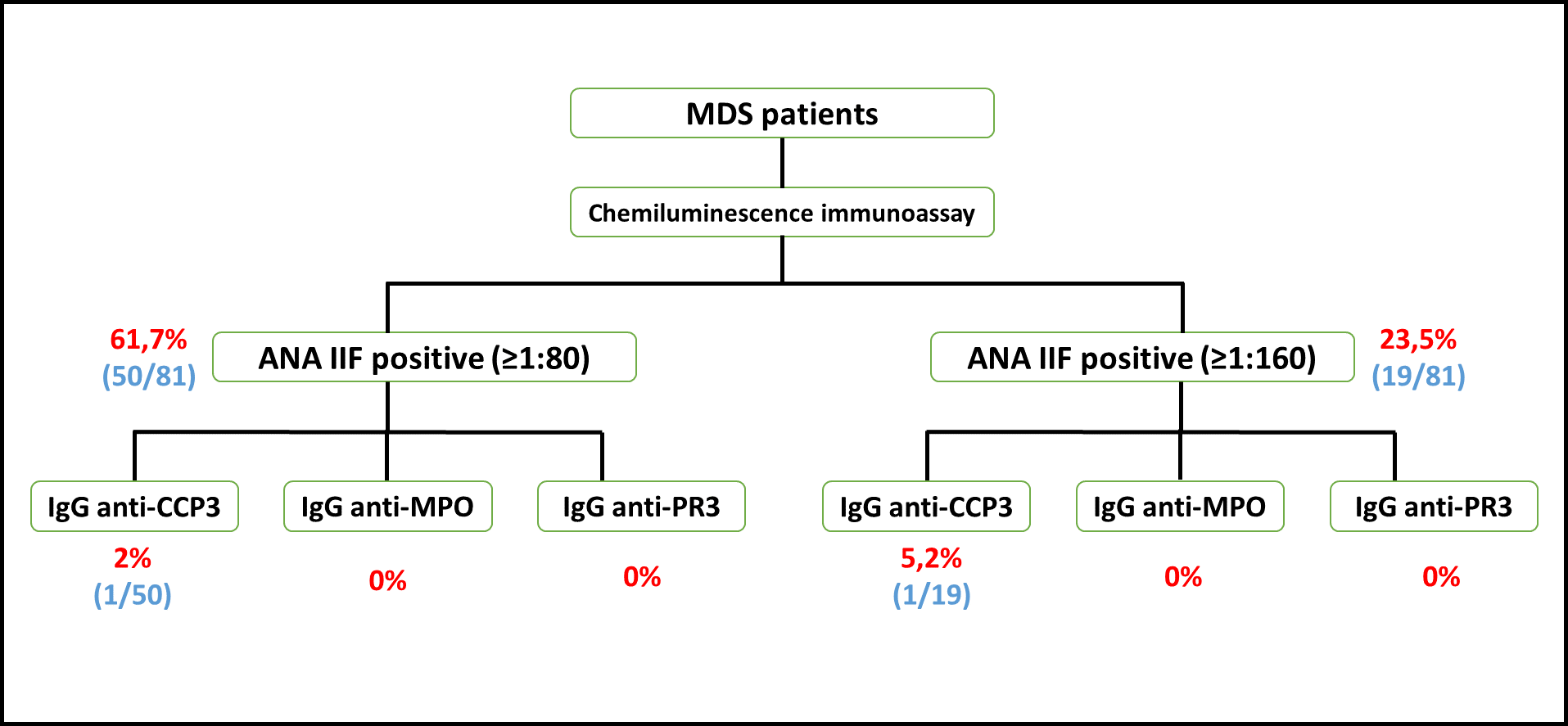


**Supplementary Figure 6. A)** Frequency of ANA≥1:160 positivity in MDS cohort according to patients ‘age. **B)** Frequency of ANA≥1:160 positivity in MDS cohort according to IPSS-R category. **C)** Frequency of ANA≥1:160 positivity in MDS cohort according to 2016 WHO classification


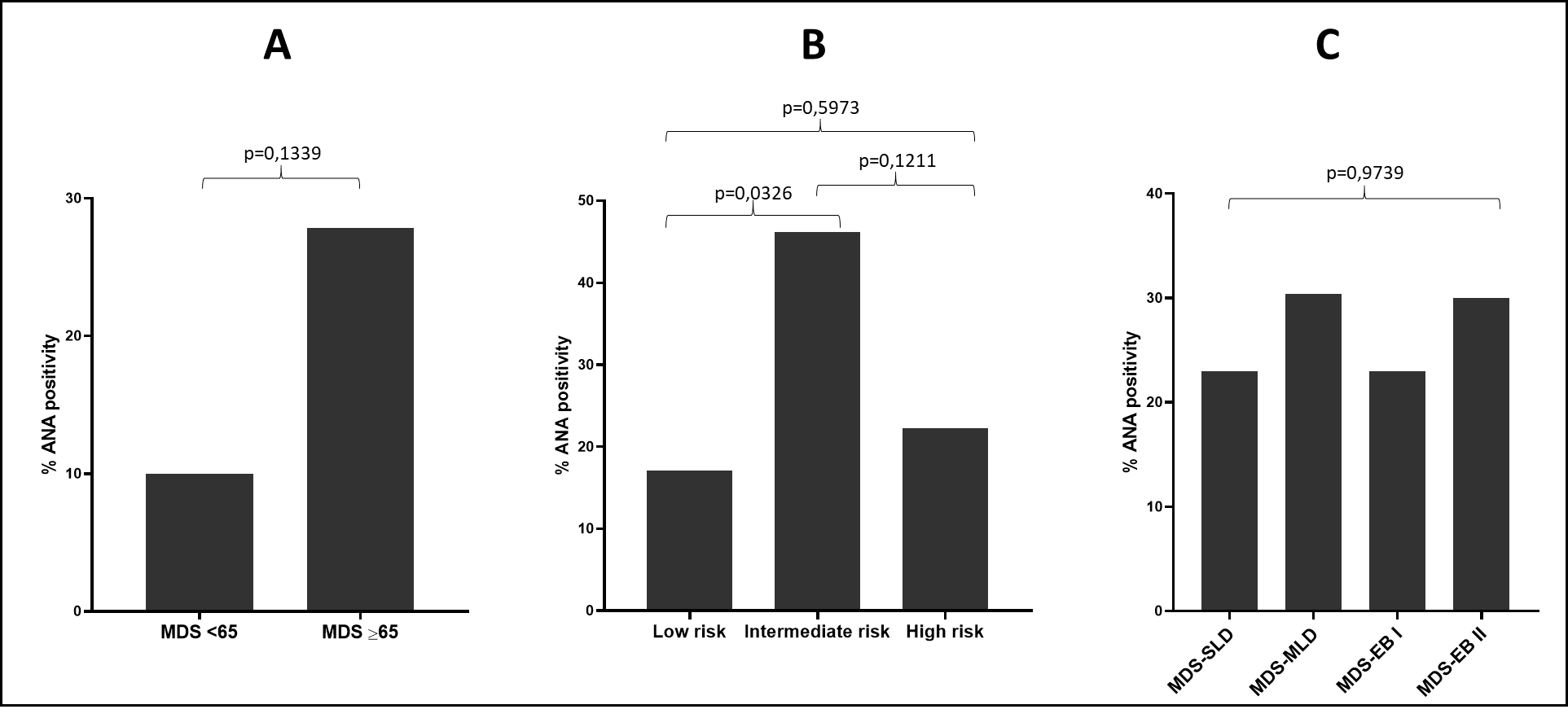


**Supplementary Figure 7.** Frequency of the most mutated genes comparing ANA ≥1:160 positive MDS patients and MDS patients with ANA negativity or ANA <1:160


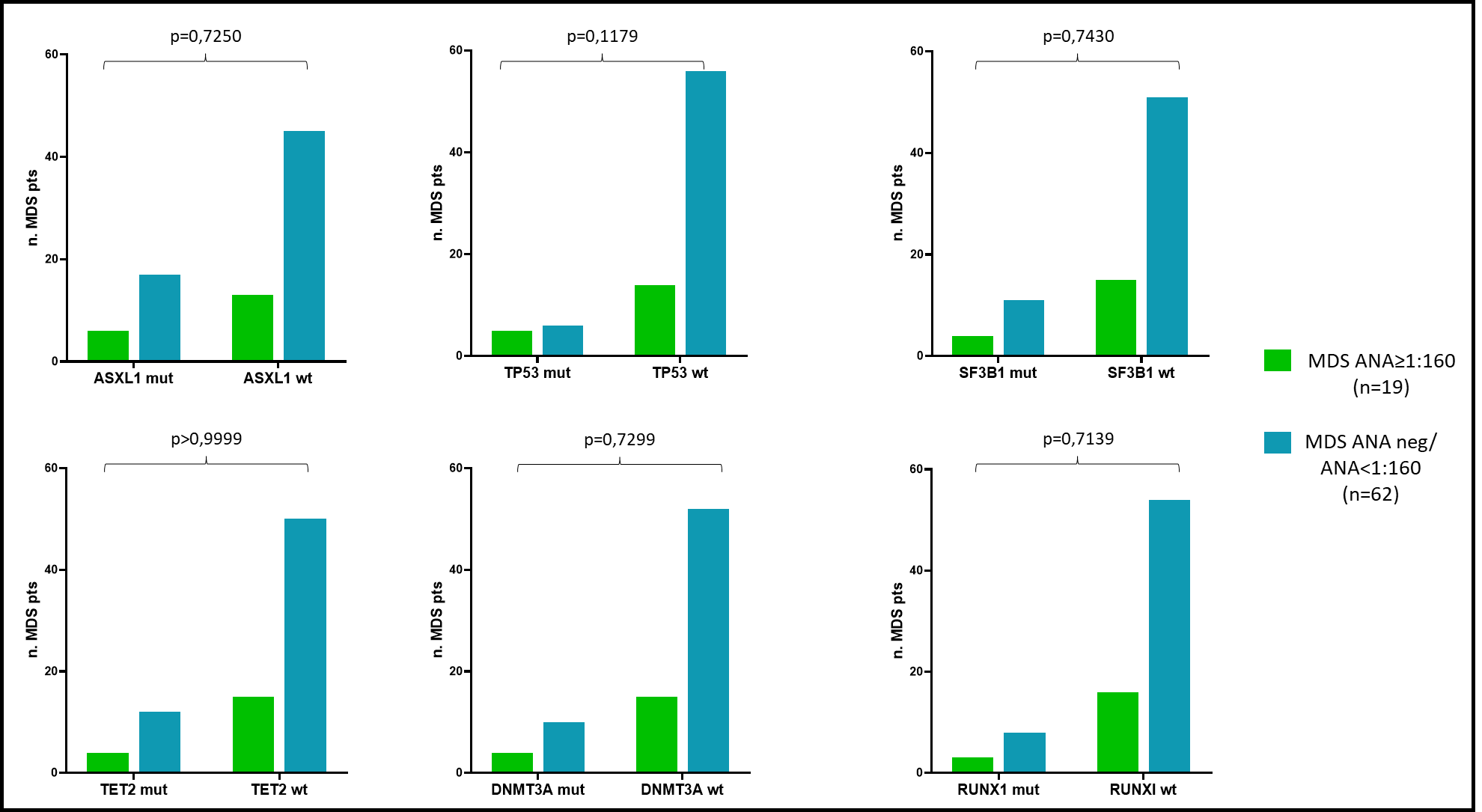

Supplement: Supplementary file 1 — Supplementary file1 (DOCX 311 KB) [file 11_2023_1773_MOESM1_ESM.docx]
